# *rac*-6-Eth­oxy-3,3a,4,9b-tetra­hydro-1,3-diphenyl-1*H*-chromeno[4,3-*c*]isoxazole-3a-carbonitrile

**DOI:** 10.1107/S160053681201906X

**Published:** 2012-05-05

**Authors:** S. Paramasivam, J. Srinivasan, P. R. Seshadri, M. Bakthadoss

**Affiliations:** a Post Graduate and Research Department of Physics, Agurchand Manmull Jain College, Chennai 600 114, India; b Department of Organic Chemistry, University of Madras, Guindy Campus, Chennai 600 025, India

## Abstract

The title compound, C_25_H_22_N_2_O_3_, with three stereogenic centres, crystallizes in a centrosymmetric space group as a racemate. The pyran ring adopts a sofa conformation and the five-membered isoxazole ring exhibits an envelope conformation. The dihedral angle between the benzene ring and the mean plane through the near coplanar atoms of the pyran ring is 10.54 (9)°. In the crystal, no significant intermolecular interactions are observed.

## Related literature

For the biological activity of the title compound, see: Rozman *et al.* (2002 ▶); Winn *et al.* (1976 ▶). For N-atom hybridization, see: Beddoes *et al.* (1986 ▶). For conformational analysis and puckering parameters, see: Cremer & Pople, (1975 ▶). For related structures, see: Kanchanadevi *et al.* (2011 ▶); Swaminathan *et al.* (2012 ▶).
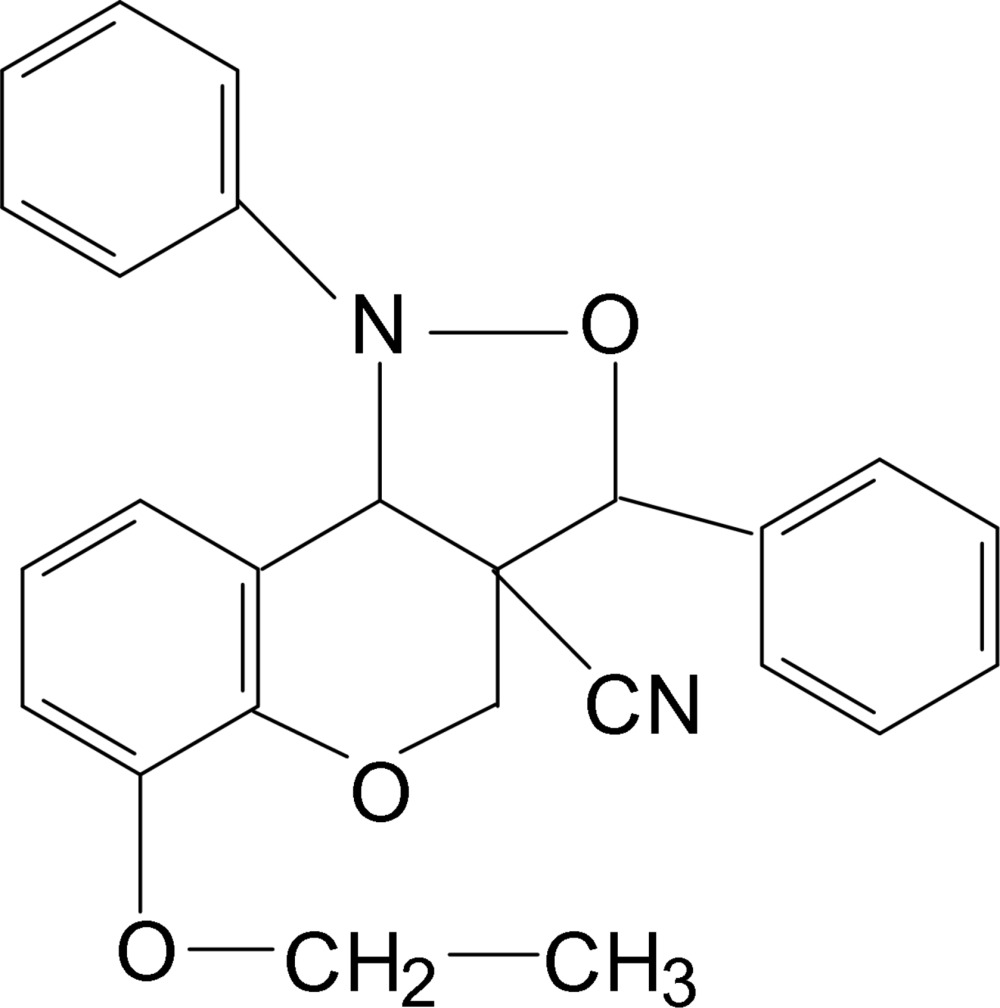


## Experimental

### 

#### Crystal data

C_25_H_22_N_2_O_3_
*M* *_r_* = 398.45
Monoclinic, 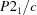

*a* = 15.2994 (9) Å
*b* = 7.5421 (5) Å
*c* = 18.7248 (12) Å
β = 107.596 (4)°
*V* = 2059.6 (2) Å^3^
*Z* = 4
Mo *K*α radiation
μ = 0.09 mm^−1^
*T* = 298 K
0.20 × 0.15 × 0.10 mm

#### Data collection

Bruker SMART APEXII area-detector diffractometer
18432 measured reflections
5109 independent reflections
2805 reflections with *I* > 2σ(*I*)
*R*_int_ = 0.029

#### Refinement

*R*[*F* ^2^ > 2σ(*F* ^2^)] = 0.051
*wR*(*F* ^2^) = 0.164
*S* = 1.04
5109 reflections
271 parameters
H-atom parameters constrained
Δρ_max_ = 0.24 e Å^−3^
Δρ_min_ = −0.18 e Å^−3^

### 

Data collection: *APEX2* (Bruker, 2008 ▶); cell refinement: *SAINT* (Bruker, 2008 ▶); data reduction: *SAINT*; program(s) used to solve structure: *SHELXS97* (Sheldrick, 2008 ▶); program(s) used to refine structure: *SHELXL97* (Sheldrick, 2008 ▶); molecular graphics: *ORTEP-3* (Farrugia, 1997 ▶) and *PLATON* (Spek, 2009 ▶); software used to prepare material for publication: *SHELXL97* (Sheldrick, 2008 ▶), *PLATON* and *publCIF* (Westrip, 2010 ▶).

## Supplementary Material

Crystal structure: contains datablock(s) I, global. DOI: 10.1107/S160053681201906X/kp2408sup1.cif

Structure factors: contains datablock(s) I. DOI: 10.1107/S160053681201906X/kp2408Isup3.hkl

Supplementary material file. DOI: 10.1107/S160053681201906X/kp2408Isup3.cml

Additional supplementary materials:  crystallographic information; 3D view; checkCIF report

## Figures and Tables

**Fig. 1.**
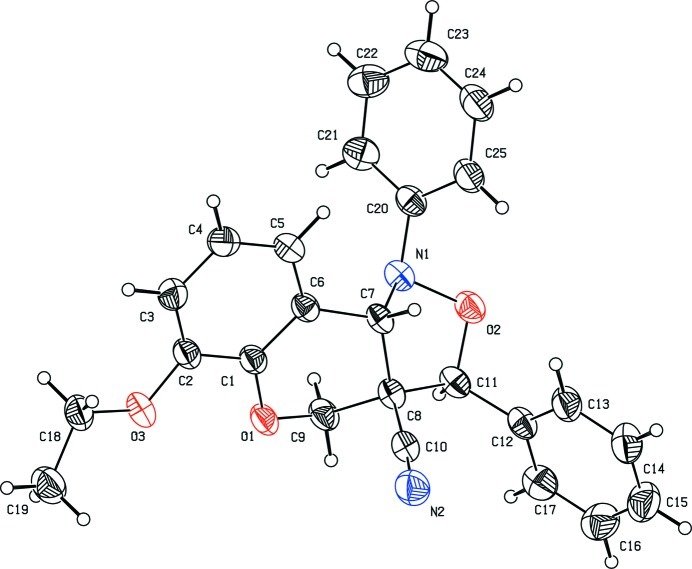
The molecular structure of the title compound, showing the atom-numbering scheme and displacement ellipsoids drawn at the 30% probability level.

## supplementary crystallographic information

### Comment

Isoxazole derivative is used for the treatment of rheumatoid arthritis (Rozman *et al.*, 2002) whereas benzopyran derivatives exhibit anti-depressant activities (Winn *et al.*, 1976). On this grounds, the title compound was chosen for X-ray structure analysis (Fig.1).

The pyran ring (O1/C1/C6—C9) adopts a sofa conformation with the puckering parameters (Cremer & Pople, 1975) being q_2_=0.426 (1) Å, q_3_=0.291 (1) Å, Q_T_=0.516 (1) Å and the five-membered isoxazole ring (N1/O2/C7/C8/C11) adopts an envelope conformation with puckering parameters (Cremer & Pople, 1975) being q_2_=0.521 (19) Å and Φ_2_=219.3 (2)°. The dihedral angle between the pyran and the benzene rings (C1—C6) is 7.68 (5)°. Also the dihedral angle between the chromeno ring (fusion of benzene and pyran rings) and isoxazole ring is 40.31 (5)°.

In the chromenoisoxazole moiety, the dihedral angle between the benzene and isoxazole ring is 36.41 (5)° and the dihedral angle between the pyran and isoxazole ring is 42.56 (6)°.

The sum of the bond angles around N1 [321.17 (39)°] indicates *sp*^3^ hybridization (Beddoes *et al.*,1986).

The geometric parameters of the title compound (Fig. 1) agree well with the reported similar structures (Kanchanadevi *et al.*, 2011; Swaminathan *et al.*, 2012).

The molecular structure is stabilized by C— H··· N intramolecular interaction and the crystal packing is stabilised by C— H··· O and C— H··· N hydrogen bonds (Table 1).

### Experimental

A mixture of (*E*)-2-((2-ethoxy-6-formylphenoxy)methyl)-3-phenylacrylonitrile (2 mmol, 0.61 g) and *N*-phenylhydroxylamine (3 mmol, 0.33 g) in ethanol (10 mL) was refluxed for 6 h. After the completion of the reaction as indicated by TLC, the reaction mixture was concentrated and the resulting crude mass was diluted with water (15 mL) and extracted with ethyl acetate (3 × 15 mL). The combined organic layer was washed with brine (3 × 15 mL) and dried over anhydrous Na_2_SO_4_, solvent was removed under reduced pressure. The crude mass was purified by column chromatography on silica gel (Acme 100–200 mesh), using ethyl acetate-hexane (1:9) to afford the pure compound as a colourless solid in 76% yield.

### Refinement

Hydrogen atoms were positioned geometrically and allowed to ride on their parent atoms, with C—H = 0.93 - 0.97 Å and *U*_iso_(H) = 1.5*U*_eq_(C) for methyl H atoms and 1.2 *U*_eq_(C) for other H atoms.

### Crystal data

**Table:**

C_25_H_22_N_2_O_3_	*F*(000) = 840
*M**_r_* = 398.45	*D*_x_ = 1.285 Mg m^−^^3^
Monoclinic, *P*2_1_/*c*	Mo *K*α radiation, λ = 0.71073 Å
Hall symbol: -P 2ybc	Cell parameters from 5109 reflections
*a* = 15.2994 (9) Å	θ = 1.4–28.3°
*b* = 7.5421 (5) Å	µ = 0.09 mm^−^^1^
*c* = 18.7248 (12) Å	*T* = 298 K
β = 107.596 (4)°	Block, colourless
*V* = 2059.6 (2) Å^3^	0.20 × 0.15 × 0.10 mm
*Z* = 4	

### Data collection

**Table:**

Bruker SMART APEXII area-detector diffractometer	2805 reflections with *I* > 2σ(*I*)
Radiation source: fine-focus sealed tube	*R*_int_ = 0.029
Graphite monochromator	θ_max_ = 28.3°, θ_min_ = 1.4°
ω and φ scans	*h* = −20→20
18432 measured reflections	*k* = −10→10
5109 independent reflections	*l* = −20→24

### Refinement

**Table:**

Refinement on *F*^2^	Primary atom site location: structure-invariant direct methods
Least-squares matrix: full	Secondary atom site location: difference Fourier map
*R*[*F*^2^ > 2σ(*F*^2^)] = 0.051	Hydrogen site location: inferred from neighbouring sites
*wR*(*F*^2^) = 0.164	H-atom parameters constrained
*S* = 1.04	*w* = 1/[σ^2^(*F*_o_^2^) + (0.0749*P*)^2^ + 0.2385*P*] where *P* = (*F*_o_^2^ + 2*F*_c_^2^)/3
5109 reflections	(Δ/σ)_max_ = 0.008
271 parameters	Δρ_max_ = 0.24 e Å^−^^3^
0 restraints	Δρ_min_ = −0.18 e Å^−^^3^

### Special details

**Table:**

Geometry. All e.s.d.'s (except the e.s.d. in the dihedral angle between two l.s. planes) are estimated using the full covariance matrix. The cell e.s.d.'s are taken into account individually in the estimation of e.s.d.'s in distances, angles and torsion angles; correlations between e.s.d.'s in cell parameters are only used when they are defined by crystal symmetry. An approximate (isotropic) treatment of cell e.s.d.'s is used for estimating e.s.d.'s involving l.s. planes.
Refinement. Refinement of *F*^2^ against ALL reflections. The weighted *R*-factor *wR* and goodness of fit *S* are based on *F*^2^, conventional *R*-factors *R* are based on *F*, with *F* set to zero for negative *F*^2^. The threshold expression of *F*^2^ > σ(*F*^2^) is used only for calculating *R*-factors(gt) *etc*. and is not relevant to the choice of reflections for refinement. *R*-factors based on *F*^2^ are statistically about twice as large as those based on *F*, and *R*- factors based on ALL data will be even larger.

### Fractional atomic coordinates and isotropic or equivalent isotropic displacement parameters (Å^2^)

**Table:**

	*x*	*y*	*z*	*U*_iso_*/*U*_eq_	
O1	0.74167 (9)	0.45965 (16)	0.72376 (7)	0.0602 (4)	
O2	0.88519 (9)	−0.03937 (17)	0.68806 (7)	0.0612 (4)	
O3	0.59365 (9)	0.65386 (17)	0.67710 (8)	0.0672 (4)	
N1	0.81401 (10)	0.0634 (2)	0.63282 (8)	0.0543 (4)	
C7	0.74962 (12)	0.0946 (2)	0.67721 (10)	0.0476 (4)	
H7	0.7257	−0.0189	0.6887	0.057*	
C8	0.81684 (12)	0.1717 (2)	0.74828 (10)	0.0514 (5)	
C1	0.67024 (13)	0.3889 (2)	0.66889 (10)	0.0502 (4)	
C6	0.67181 (12)	0.2174 (2)	0.64144 (9)	0.0483 (4)	
C12	0.94613 (12)	−0.0419 (3)	0.82161 (11)	0.0556 (5)	
C11	0.90766 (13)	0.0711 (3)	0.75323 (11)	0.0561 (5)	
H11	0.9542	0.1573	0.7500	0.067*	
C20	0.77892 (12)	−0.0486 (3)	0.56829 (11)	0.0548 (5)	
C9	0.82649 (14)	0.3691 (2)	0.73450 (11)	0.0600 (5)	
H9A	0.8727	0.4202	0.7770	0.072*	
H9B	0.8466	0.3845	0.6905	0.072*	
C2	0.59079 (13)	0.4927 (2)	0.64301 (10)	0.0542 (5)	
C5	0.59528 (13)	0.1560 (3)	0.58543 (10)	0.0568 (5)	
H5	0.5957	0.0426	0.5660	0.068*	
C17	1.02141 (13)	0.0150 (3)	0.87856 (12)	0.0701 (6)	
H17	1.0493	0.1221	0.8738	0.084*	
C10	0.78370 (14)	0.1491 (3)	0.81384 (11)	0.0583 (5)	
C3	0.51685 (14)	0.4285 (3)	0.58771 (11)	0.0620 (5)	
H3	0.4646	0.4981	0.5695	0.074*	
C18	0.50900 (15)	0.7498 (3)	0.66297 (12)	0.0695 (6)	
H18A	0.4604	0.6702	0.6658	0.083*	
H18B	0.4922	0.8014	0.6132	0.083*	
C21	0.75973 (14)	0.0310 (3)	0.49899 (12)	0.0689 (6)	
H21	0.7702	0.1517	0.4956	0.083*	
C4	0.51968 (14)	0.2608 (3)	0.55889 (11)	0.0645 (5)	
H4	0.4695	0.2189	0.5209	0.077*	
N2	0.75559 (15)	0.1391 (3)	0.86341 (11)	0.0825 (6)	
C13	0.90569 (15)	−0.2018 (3)	0.83037 (13)	0.0706 (6)	
H13	0.8547	−0.2422	0.7928	0.085*	
C24	0.72930 (16)	−0.3275 (3)	0.50900 (16)	0.0823 (7)	
H24	0.7191	−0.4485	0.5120	0.099*	
C25	0.76250 (14)	−0.2284 (3)	0.57391 (13)	0.0674 (6)	
H25	0.7736	−0.2816	0.6206	0.081*	
C14	0.94057 (18)	−0.3005 (3)	0.89414 (14)	0.0833 (7)	
H14	0.9130	−0.4073	0.8997	0.100*	
C23	0.71127 (16)	−0.2466 (4)	0.43976 (15)	0.0866 (8)	
H23	0.6897	−0.3139	0.3964	0.104*	
C22	0.72498 (16)	−0.0680 (4)	0.43459 (13)	0.0818 (7)	
H22	0.7109	−0.0137	0.3879	0.098*	
C19	0.52145 (18)	0.8905 (3)	0.71959 (13)	0.0847 (7)	
H19A	0.4654	0.9562	0.7107	0.127*	
H19B	0.5696	0.9688	0.7164	0.127*	
H19C	0.5374	0.8383	0.7686	0.127*	
C15	1.01620 (18)	−0.2420 (4)	0.94988 (14)	0.0872 (7)	
H15	1.0403	−0.3102	0.9927	0.105*	
C16	1.05552 (16)	−0.0852 (4)	0.94225 (14)	0.0865 (7)	
H16	1.1060	−0.0449	0.9804	0.104*	

### Atomic displacement parameters (Å^2^)

**Table:**

	*U*^11^	*U*^22^	*U*^33^	*U*^12^	*U*^13^	*U*^23^
O1	0.0672 (8)	0.0368 (7)	0.0725 (8)	0.0015 (7)	0.0151 (7)	−0.0087 (6)
O2	0.0582 (7)	0.0561 (8)	0.0708 (8)	0.0089 (7)	0.0220 (6)	−0.0094 (7)
O3	0.0757 (9)	0.0444 (8)	0.0851 (9)	0.0107 (7)	0.0299 (7)	−0.0058 (7)
N1	0.0549 (9)	0.0488 (9)	0.0627 (9)	−0.0006 (8)	0.0231 (7)	−0.0060 (8)
C7	0.0520 (10)	0.0342 (9)	0.0616 (10)	−0.0033 (8)	0.0244 (8)	−0.0045 (8)
C8	0.0555 (10)	0.0395 (10)	0.0610 (10)	0.0004 (9)	0.0204 (8)	−0.0017 (8)
C1	0.0605 (11)	0.0397 (10)	0.0534 (10)	−0.0001 (9)	0.0218 (9)	−0.0007 (8)
C6	0.0554 (10)	0.0399 (10)	0.0545 (10)	−0.0027 (9)	0.0240 (8)	−0.0013 (8)
C12	0.0487 (10)	0.0470 (11)	0.0731 (12)	0.0038 (9)	0.0214 (9)	−0.0080 (10)
C11	0.0546 (11)	0.0458 (11)	0.0710 (12)	−0.0044 (9)	0.0238 (9)	−0.0098 (10)
C20	0.0519 (10)	0.0526 (12)	0.0648 (11)	0.0029 (9)	0.0251 (9)	−0.0079 (10)
C9	0.0638 (12)	0.0409 (11)	0.0730 (12)	−0.0069 (10)	0.0176 (10)	−0.0079 (9)
C2	0.0712 (12)	0.0392 (10)	0.0593 (10)	0.0030 (10)	0.0305 (10)	0.0009 (9)
C5	0.0623 (12)	0.0500 (11)	0.0601 (11)	−0.0002 (10)	0.0215 (9)	−0.0089 (9)
C17	0.0510 (11)	0.0743 (15)	0.0850 (15)	0.0001 (11)	0.0207 (11)	−0.0111 (13)
C10	0.0663 (12)	0.0445 (11)	0.0633 (12)	0.0067 (10)	0.0185 (10)	−0.0027 (10)
C3	0.0659 (12)	0.0612 (13)	0.0592 (11)	0.0117 (11)	0.0195 (10)	0.0024 (10)
C18	0.0842 (15)	0.0556 (13)	0.0758 (13)	0.0181 (12)	0.0346 (11)	0.0065 (11)
C21	0.0695 (13)	0.0741 (15)	0.0702 (13)	−0.0071 (12)	0.0318 (11)	−0.0062 (12)
C4	0.0642 (12)	0.0679 (14)	0.0581 (11)	0.0047 (11)	0.0135 (9)	−0.0080 (10)
N2	0.1075 (15)	0.0767 (14)	0.0739 (12)	0.0162 (12)	0.0432 (11)	0.0031 (10)
C13	0.0691 (13)	0.0496 (13)	0.0844 (15)	0.0020 (11)	0.0100 (11)	−0.0020 (11)
C24	0.0709 (14)	0.0604 (14)	0.1057 (19)	0.0073 (12)	0.0118 (13)	−0.0213 (14)
C25	0.0668 (13)	0.0516 (12)	0.0803 (13)	0.0061 (11)	0.0170 (10)	−0.0087 (11)
C14	0.0882 (17)	0.0614 (14)	0.0978 (17)	0.0092 (13)	0.0241 (14)	0.0120 (13)
C23	0.0663 (14)	0.105 (2)	0.0869 (18)	0.0002 (15)	0.0207 (12)	−0.0399 (17)
C22	0.0761 (15)	0.105 (2)	0.0679 (14)	−0.0043 (15)	0.0270 (11)	−0.0103 (14)
C19	0.1040 (18)	0.0717 (15)	0.0895 (16)	0.0167 (14)	0.0460 (14)	−0.0053 (13)
C15	0.0762 (16)	0.093 (2)	0.0865 (17)	0.0249 (16)	0.0158 (13)	0.0133 (15)
C16	0.0588 (13)	0.110 (2)	0.0824 (16)	0.0111 (15)	0.0096 (12)	−0.0049 (16)

### Geometric parameters (Å, º)

**Table:**

O1—C1	1.362 (2)	C17—C16	1.375 (3)
O1—C9	1.426 (2)	C17—H17	0.9300
O2—C11	1.431 (2)	C10—N2	1.137 (2)
O2—N1	1.4748 (19)	C3—C4	1.381 (3)
O3—C2	1.368 (2)	C3—H3	0.9300
O3—C18	1.437 (2)	C18—C19	1.471 (3)
N1—C20	1.438 (2)	C18—H18A	0.9700
N1—C7	1.487 (2)	C18—H18B	0.9700
C7—C6	1.497 (2)	C21—C22	1.381 (3)
C7—C8	1.529 (2)	C21—H21	0.9300
C7—H7	0.9800	C4—H4	0.9300
C8—C10	1.473 (3)	C13—C14	1.371 (3)
C8—C9	1.526 (3)	C13—H13	0.9300
C8—C11	1.561 (3)	C24—C23	1.383 (4)
C1—C6	1.395 (2)	C24—C25	1.386 (3)
C1—C2	1.403 (3)	C24—H24	0.9300
C6—C5	1.394 (2)	C25—H25	0.9300
C12—C17	1.380 (3)	C14—C15	1.376 (3)
C12—C13	1.388 (3)	C14—H14	0.9300
C12—C11	1.502 (3)	C23—C22	1.371 (4)
C11—H11	0.9800	C23—H23	0.9300
C20—C21	1.378 (3)	C22—H22	0.9300
C20—C25	1.389 (3)	C19—H19A	0.9600
C9—H9A	0.9700	C19—H19B	0.9600
C9—H9B	0.9700	C19—H19C	0.9600
C2—C3	1.370 (3)	C15—C16	1.354 (4)
C5—C4	1.365 (3)	C15—H15	0.9300
C5—H5	0.9300	C16—H16	0.9300
			
C1—O1—C9	114.04 (14)	C16—C17—H17	119.7
C11—O2—N1	103.20 (12)	C12—C17—H17	119.7
C2—O3—C18	117.52 (16)	N2—C10—C8	176.6 (2)
C20—N1—O2	106.84 (13)	C2—C3—C4	120.16 (19)
C20—N1—C7	114.86 (14)	C2—C3—H3	119.9
O2—N1—C7	99.47 (12)	C4—C3—H3	119.9
N1—C7—C6	114.78 (14)	O3—C18—C19	108.52 (18)
N1—C7—C8	99.30 (13)	O3—C18—H18A	110.0
C6—C7—C8	112.86 (14)	C19—C18—H18A	110.0
N1—C7—H7	109.8	O3—C18—H18B	110.0
C6—C7—H7	109.8	C19—C18—H18B	110.0
C8—C7—H7	109.8	H18A—C18—H18B	108.4
C10—C8—C9	109.21 (15)	C20—C21—C22	120.2 (2)
C10—C8—C7	111.77 (15)	C20—C21—H21	119.9
C9—C8—C7	107.33 (15)	C22—C21—H21	119.9
C10—C8—C11	114.76 (15)	C5—C4—C3	120.78 (19)
C9—C8—C11	110.76 (15)	C5—C4—H4	119.6
C7—C8—C11	102.66 (14)	C3—C4—H4	119.6
O1—C1—C6	122.82 (16)	C14—C13—C12	120.3 (2)
O1—C1—C2	117.10 (16)	C14—C13—H13	119.8
C6—C1—C2	119.98 (17)	C12—C13—H13	119.8
C5—C6—C1	118.78 (17)	C23—C24—C25	120.0 (2)
C5—C6—C7	120.29 (16)	C23—C24—H24	120.0
C1—C6—C7	120.58 (16)	C25—C24—H24	120.0
C17—C12—C13	118.5 (2)	C24—C25—C20	119.1 (2)
C17—C12—C11	120.17 (19)	C24—C25—H25	120.4
C13—C12—C11	121.32 (18)	C20—C25—H25	120.4
O2—C11—C12	109.16 (15)	C13—C14—C15	120.2 (2)
O2—C11—C8	104.61 (14)	C13—C14—H14	119.9
C12—C11—C8	116.01 (15)	C15—C14—H14	119.9
O2—C11—H11	108.9	C22—C23—C24	120.5 (2)
C12—C11—H11	108.9	C22—C23—H23	119.7
C8—C11—H11	108.9	C24—C23—H23	119.7
C21—C20—C25	120.29 (19)	C23—C22—C21	119.7 (2)
C21—C20—N1	117.04 (18)	C23—C22—H22	120.1
C25—C20—N1	122.65 (18)	C21—C22—H22	120.1
O1—C9—C8	111.13 (15)	C18—C19—H19A	109.5
O1—C9—H9A	109.4	C18—C19—H19B	109.5
C8—C9—H9A	109.4	H19A—C19—H19B	109.5
O1—C9—H9B	109.4	C18—C19—H19C	109.5
C8—C9—H9B	109.4	H19A—C19—H19C	109.5
H9A—C9—H9B	108.0	H19B—C19—H19C	109.5
O3—C2—C3	124.70 (18)	C16—C15—C14	119.9 (2)
O3—C2—C1	115.63 (17)	C16—C15—H15	120.0
C3—C2—C1	119.67 (17)	C14—C15—H15	120.0
C4—C5—C6	120.53 (18)	C15—C16—C17	120.5 (2)
C4—C5—H5	119.7	C15—C16—H16	119.7
C6—C5—H5	119.7	C17—C16—H16	119.7
C16—C17—C12	120.6 (2)		
			
C11—O2—N1—C20	−172.76 (14)	O2—N1—C20—C25	40.8 (2)
C11—O2—N1—C7	−53.05 (15)	C7—N1—C20—C25	−68.5 (2)
C20—N1—C7—C6	−73.56 (19)	C1—O1—C9—C8	−54.2 (2)
O2—N1—C7—C6	172.81 (13)	C10—C8—C9—O1	−57.5 (2)
C20—N1—C7—C8	165.83 (15)	C7—C8—C9—O1	63.88 (19)
O2—N1—C7—C8	52.20 (14)	C11—C8—C9—O1	175.21 (14)
N1—C7—C8—C10	−156.53 (15)	C18—O3—C2—C3	10.7 (3)
C6—C7—C8—C10	81.46 (18)	C18—O3—C2—C1	−168.42 (16)
N1—C7—C8—C9	83.74 (16)	O1—C1—C2—O3	−1.0 (2)
C6—C7—C8—C9	−38.3 (2)	C6—C1—C2—O3	175.48 (15)
N1—C7—C8—C11	−33.04 (16)	O1—C1—C2—C3	179.81 (16)
C6—C7—C8—C11	−155.05 (14)	C6—C1—C2—C3	−3.7 (3)
C9—O1—C1—C6	18.2 (2)	C1—C6—C5—C4	−1.1 (3)
C9—O1—C1—C2	−165.40 (15)	C7—C6—C5—C4	172.18 (17)
O1—C1—C6—C5	179.70 (16)	C13—C12—C17—C16	0.3 (3)
C2—C1—C6—C5	3.4 (3)	C11—C12—C17—C16	178.19 (19)
O1—C1—C6—C7	6.5 (3)	O3—C2—C3—C4	−177.50 (18)
C2—C1—C6—C7	−169.83 (15)	C1—C2—C3—C4	1.6 (3)
N1—C7—C6—C5	80.0 (2)	C2—O3—C18—C19	164.34 (17)
C8—C7—C6—C5	−167.23 (16)	C25—C20—C21—C22	−0.7 (3)
N1—C7—C6—C1	−106.92 (18)	N1—C20—C21—C22	−179.33 (18)
C8—C7—C6—C1	5.9 (2)	C6—C5—C4—C3	−1.0 (3)
N1—O2—C11—C12	155.50 (14)	C2—C3—C4—C5	0.8 (3)
N1—O2—C11—C8	30.73 (17)	C17—C12—C13—C14	−0.4 (3)
C17—C12—C11—O2	137.73 (18)	C11—C12—C13—C14	−178.3 (2)
C13—C12—C11—O2	−44.4 (2)	C23—C24—C25—C20	−1.2 (3)
C17—C12—C11—C8	−104.5 (2)	C21—C20—C25—C24	2.0 (3)
C13—C12—C11—C8	73.4 (2)	N1—C20—C25—C24	−179.50 (18)
C10—C8—C11—O2	123.37 (17)	C12—C13—C14—C15	−0.2 (4)
C9—C8—C11—O2	−112.41 (17)	C25—C24—C23—C22	−0.7 (4)
C7—C8—C11—O2	1.90 (18)	C24—C23—C22—C21	2.0 (4)
C10—C8—C11—C12	3.1 (2)	C20—C21—C22—C23	−1.3 (3)
C9—C8—C11—C12	127.29 (17)	C13—C14—C15—C16	1.0 (4)
C7—C8—C11—C12	−118.40 (17)	C14—C15—C16—C17	−1.1 (4)
O2—N1—C20—C21	−140.65 (17)	C12—C17—C16—C15	0.5 (3)
C7—N1—C20—C21	110.10 (19)		

### Hydrogen-bond geometry (Å, º)

**Table:**

*D*—H···*A*	*D*—H	H···*A*	*D*···*A*	*D*—H···*A*
C9—H9*B*···N1	0.97	2.64	2.960 (2)	100
C25—H25···O1^i^	0.93	2.89	3.748 (3)	154
C23—H23···N2^ii^	0.93	2.79	3.443 (3)	128

Symmetry codes: (i) *x*, *y*−1, *z*; (ii) *x*, −*y*−1/2, *z*−1/2.
